# Challenges in Diagnosing Juxt-Articular Osteoid Osteoma of the Talus: A Case Report

**DOI:** 10.7759/cureus.41068

**Published:** 2023-06-28

**Authors:** Dimitrios G Economopoulos, Themistoklis Floros, Panagiotis Mandrekas, George C Babis, Vasileios S Nikolaou

**Affiliations:** 1 2nd Department of Orthopaedics, National and Kapodistrian University of Athens School of Medicine, Athens, GRC

**Keywords:** orthopaedic oncology, osteoid osteoma, talus tumour, ankle sprains, computed tomography (ct )

## Abstract

Osteoid osteomas of the talus are rarely seen. They can easily be misdiagnosed. In this case report, we present a 21-year-old man with an osteoid osteoma in the talar neck whose pain onset coincided with an ankle injury. The latter was deemed a misleading factor when making a diagnosis. Eventually, the patient was treated with surgical excision of the osteoid osteoma. The gap that resulted after the excision was filled with an autologous bone graft. A year after his operation, the patient returned to his daily activities and remained pain-free. A high index of suspicion and an appropriate imaging examination are mandated for the early diagnosis of such entities.

## Introduction

Osteoid osteomas are benign, self-limited, osteogenic bone tumours commonly found in the diaphysis and metaphysis of long bones. The proximal femur is the most distinctive site where osteoid osteomas occur, followed by the humerus, tibia, phalanges, and vertebral arch [1].

Even though osteoid osteomas are well identified on radiographs as radiolucent lesions, computed tomography is considered the diagnostic modality of choice for their diagnosis. The presence of a nidus is a typical finding in the former. Moreover, an abundance of bone oedema around these lesions is evident in MRI scans [1].

Depending on the location of the nidus, osteoid osteomas may be periosteal, intracortical, spongiosal, or subarticular [1]. Their diameter is usually less than 1 cm [2]. Even though some remain asymptomatic, most complain of regional nocturnal pain alleviated with non-steroidal anti-inflammatory drugs (NSAIDs) or aspirin [1].

The occurrence of osteoid osteomas in other sites is rare. Hence, when they present in atypical locations, they can be easily misdiagnosed, neglected, or treated with delay. In this case report, we present a 21-year-old man with an osteoid osteoma of the talus.

## Case presentation

**Figure 1:**
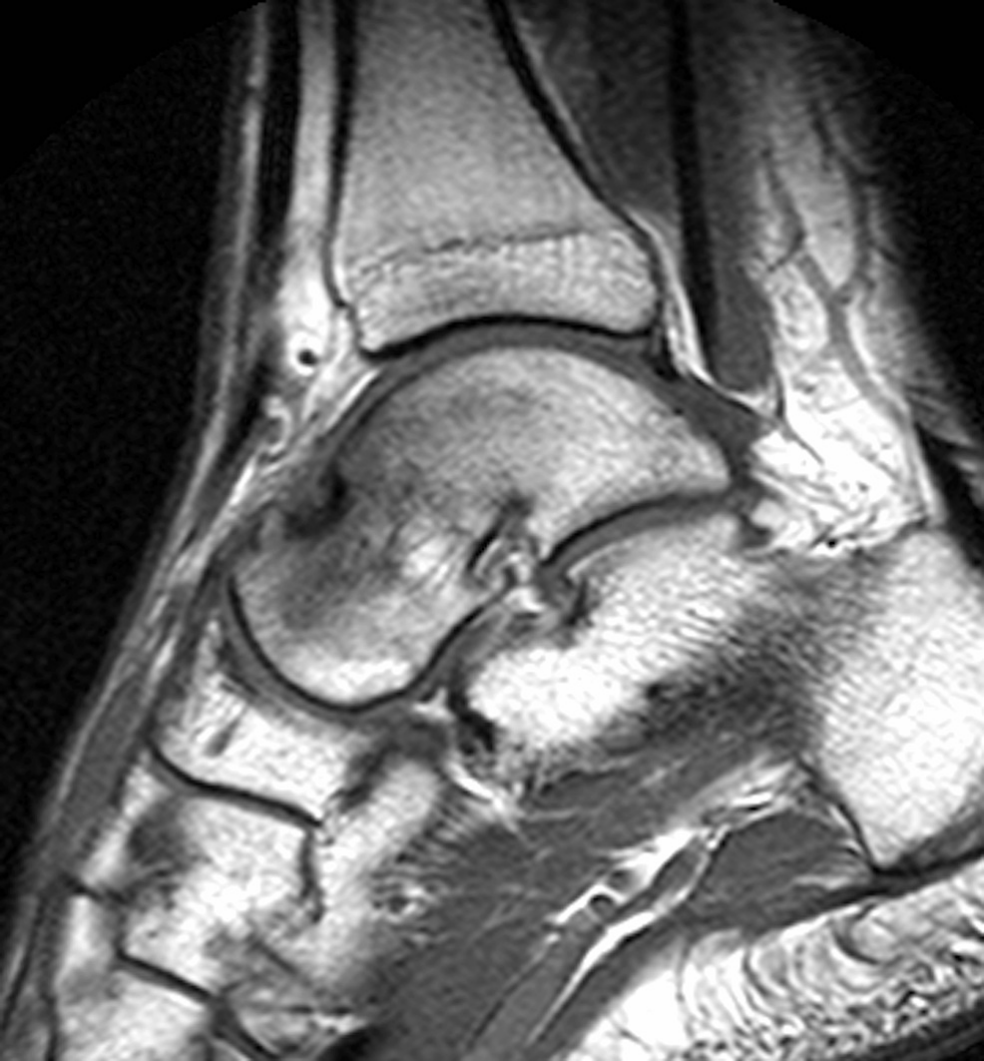
Magnetic resonance images indicated bone oedema and concomitant soft tissue inflammation at the talar neck.

A 21-year-old man visited our outpatient department complaining of chronic pain in his right ankle. He described that his symptoms started two years ago when he fell down the stairs and sustained an ankle injury. Two months later, his pain did not improve. He, therefore, sought orthopaedic consultation. The orthopaedic surgeon who initially assessed him suggested that, apart from plain radiographs, further investigation was needed and asked for an MRI scan. The latter revealed bone oedema around the talar neck and concomitant soft tissue inflammation (Figure 1).

**Figure 2:**
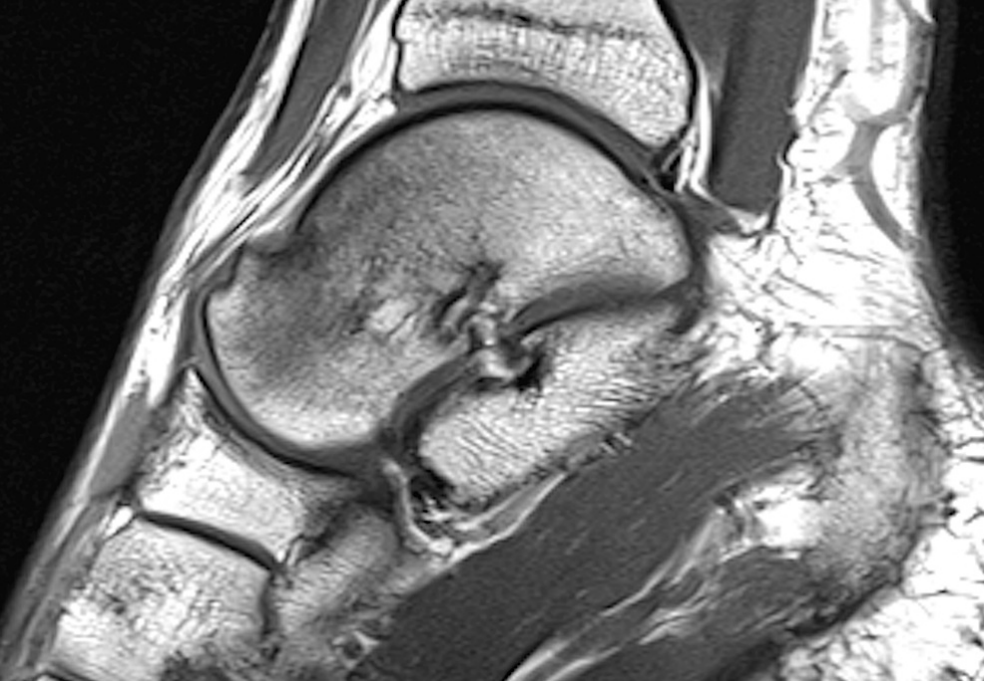
A second MRI scan, two months after his symptoms presented, showed no improvement compared to the initial findings.

His symptoms were addressed with an intra-articular corticosteroid injection, whereas NSAIDs were prescribed as supplemental pain management. Furthermore, the patient was instructed to mobilise and avoid weight bearing on the right lower extremity until further notice. Even though his symptoms improved over the first two months, the pain eventually recurred. A second MRI scan was scheduled. Its images showed that the bone marrow and soft tissue oedema located anterior to the tibiotalar joint still remained (Figure 2).

A differential diagnosis indicated anterior ankle impingement syndrome as the most probable cause of pain. Consequently, the patient received a second intra-articular corticosteroid injection and NSAIDs and remained pain-free for about a year until he reinjured his ankle. He was treated initially with rest, ice, compression, elevation (RICE), and occasional consumption of analgesics.

**Figure 3:**
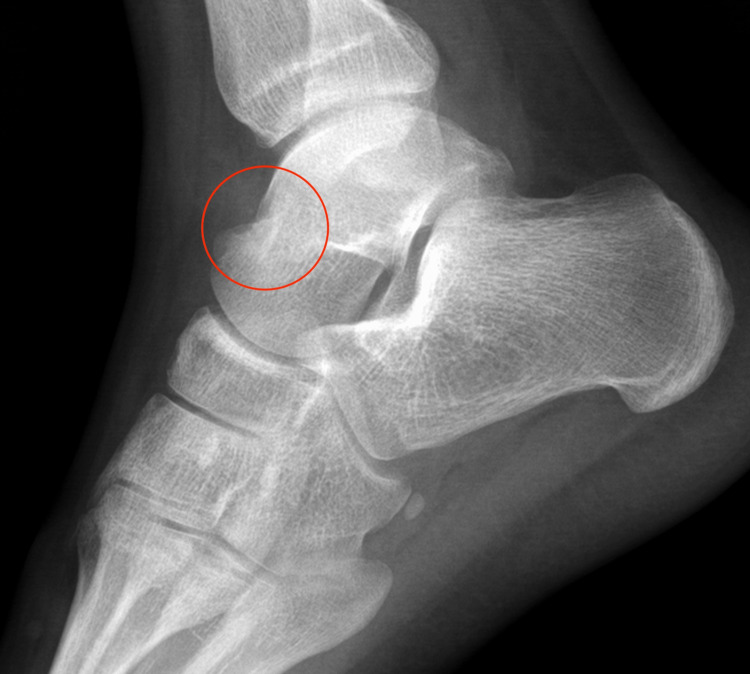
The patient's review with plain radiographs a year after the onset of his symptoms. The presence of a nidus is evident.

**Figure 4:**
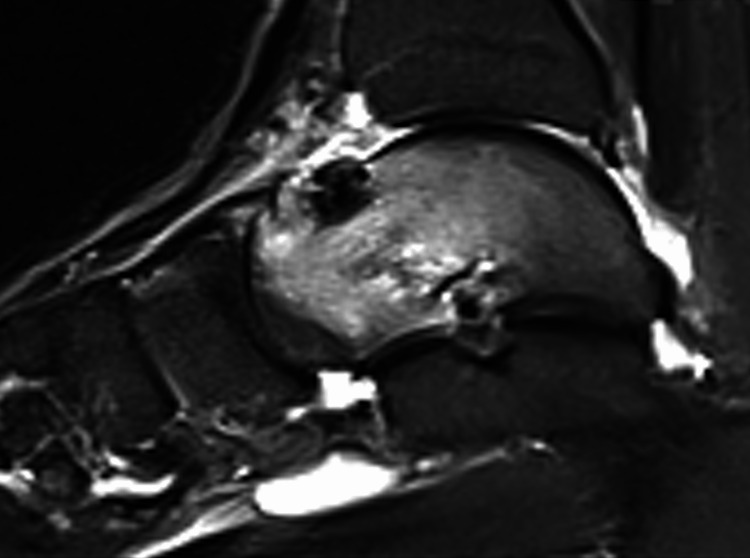
The patient's review with an MRI scan a year after the onset of his symptoms. Apart from the osteoid osteoma-related nidus, a fracture line spanning from the anterosuperior to the posteroinferior part of the talar neck is also evident.

Since his pain persisted, a third MRI scan of the ankle was advised. Bone marrow oedema was evident once again. Moreover, the MRI indicated a fracture line spanning from the anterosuperior to the posteroinferior part of the talar neck and a nidus in the talar neck. In contrast, plain radiographs showed no fracture of the talus (Figures 3-4).

A scheme of intra-articular corticosteroid injections commenced that kept the patient pain-free for a short period of time. However, after a few months, weight-bearing became gradually painful, and a progressively worsening nocturnal pain developed in the ankle.

**Figure 5:**
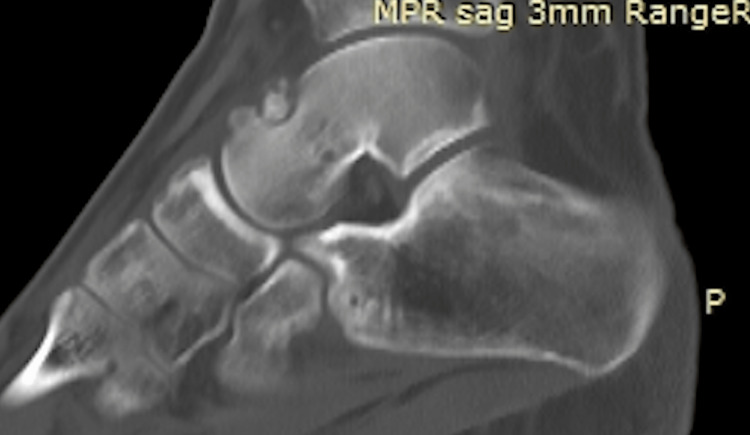
An image from a CT scan demonstrating an osteoid osteoma of the talar neck.

Two years after the onset of his symptoms, the patient was referred to our clinic for further assessment. A CT scan of his right ankle verified the presence of an osteoid osteoma in the superior surface of the talar neck (Figure 5).

The patient was given aspirin for pain management and agreed to undergo surgical excision of the osteoid osteoma.

**Figure 6:**
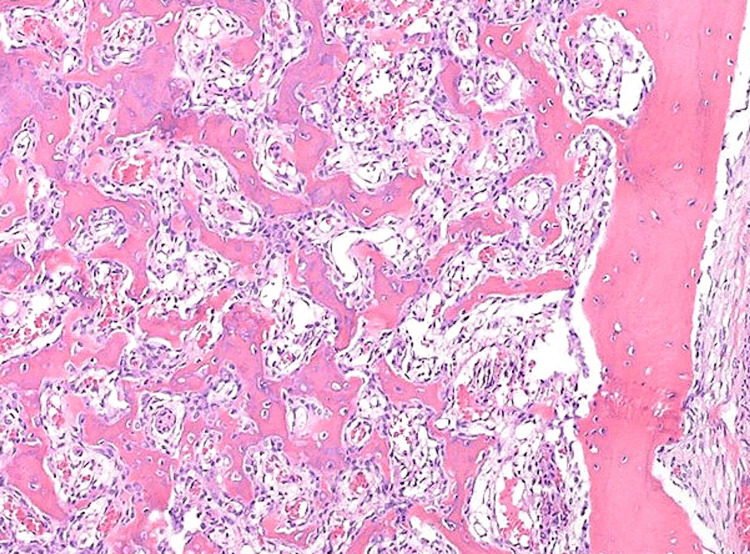
A typical microscopic image of osteoid osteoma is seen. A well-demarcated formation where osteoblastic rimming is evident in the nidus and an abundance of sclerotic bone on the periphery.

In the operation theatre, under spinal anaesthesia, the patient was positioned supine and a thigh tourniquet was applied. His right leg was prepped and draped, and then the tourniquet was inflated. A dorsal approach to the ankle was used. With the patient supine, a skin incision was made over the anterior ankle and lateral to the tibialis anterior. After the extensor retinaculum was incised, an interval between the tibialis anterior and the extensor hallucis longus was used to gain access to the ankle joint. A well-defined mahogany-coloured lesion with an approximate size of 0.9x0.8x.07 cm was then identified and curetted uneventfully. The specimen was sent to pathology for examination. The tumour bed was debrided, and the gap was filled with an autologous bone graft harvested from the distal third of the tibial diaphysis. The wound closure was performed in a standard fashion. The histopathology report confirmed that the lesion was indeed an osteoid osteoma (Figure 6).

**Figure 7:**
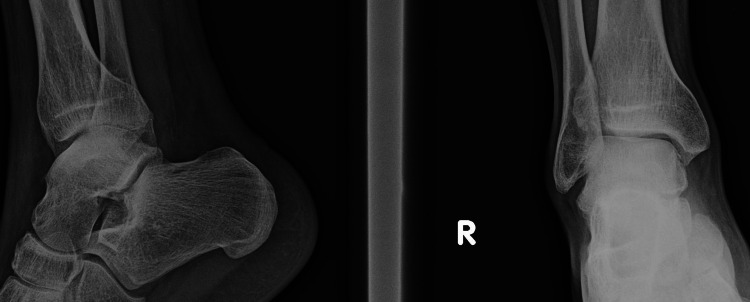
Plain radiographs a year after the excision. The patient has fully recuperated and returned uneventfully to his daily activities and amateur sports.

His rehabilitation was uneventful. On his six-month follow-up, the patient reported that his pain had fully resolved a week after his operation and remained pain-free. A year after his operation, the patient was reviewed. He had fully recovered, returned to his daily activities, and reported no discomfort when participating in sports (Figure 7).

## Discussion

Osteoid osteomas were first described by Jaffe in 1935 [3]. They represent 10% of all benign bone tumours. The differential diagnosis of osteoid osteoma includes osteoblastoma, stress fracture, osteomyelitis, and Ewing's sarcoma.

According to the Enneking classification, they are latent benign tumours that do not spread locally and have a low potential to become malignant [4]. Long bones such as the femur and tibia are common sites where osteoid osteomas develop, whereas the foot is less commonly affected (2%-10%) [5]. When this happens, osteoid osteomas develop in the hindfoot and midfoot. More specifically, in the talus and the calcaneus. Furthermore, when they do present in the talus, 97% are located in the talar neck [6]. Typically, osteoid osteomas present as central nodules of woven bone and osteoid. Histologically, the nidus and the reactive bone surrounding it are well-demarcated from each other. The nidus contains uniform osteoid seams of immature osteoid trabeculae with prominent osteoblastic rimming and a vascularised stroma. Uniform, plump osteoblasts with regularly shaped nuclei and abundant cytoplasm are also present. The reactive zone is a well-defined region of thickened bone and fibrovascular tissue around the sclerotic border [7]. The majority of foot osteoid osteomas exhibit minimal periosteal reaction and are either cancellous or subperiosteal. Osteoid osteomas and osteoblastomas are histologically identical. However, the nidus size in osteoblastomas is greater than 2 cm [8].

Pain is the most common symptom seen in osteoid osteoma patients. It is described as blunt, constant, and progressive and deteriorates at night and after alcohol consumption. Its presentation is associated with the increased number and size of unmyelinated nerve fibres within the nidus. Moreover, it is linked with the increased concentration of prostaglandins PGE2 and PGI1 and the expression of cyclooxygenases (COX-1 and COX-2) in osteoid osteoma tissue [9]. When osteoid osteomas are adjacent to a joint, oedema, erythema, tenderness, contracture, limping, and muscle atrophy may occur. Therefore, differentiating them from arthritis may be a difficult task. When the hands are affected, painless swelling may also be noted. Moreover, postural scoliosis and paravertebral muscle spasms may present when the spine is involved [10].

Radiographic assessment with plain X-rays indicates an intensely reactive bone around a small, round, radiolucent nidus. This sclerotic area around the nidus may differ depending on the tumour’s location. For example, in subperiosteal osteoid osteomas, this reactive zone is barely visible, thus making it difficult to tell them apart from soft tissue lesions. The detection rate of osteoid osteomas with plain radiographs is only 66% [11]. Thin-slice CT is the imaging method of choice for the diagnosis of osteoid osteomas. It offers valuable information about the location and size of the nidus and can detect osteoid osteomas of the foot and hand in 96.5% of cases [5,11]. The presence of curvilinear low-density grooves in the surrounding bone is a CT finding seen often in more than 80% of osteoid osteoma patients [12]. Bone scintigraphy is highly sensitive for diagnosing osteoid osteomas [13]. It offers a classic finding known as the 'double density sign'. The former depicts an intensely hot area of focal uptake at the nidus, combined with low uptake in the reactive zone [14]. Conversely, the investigation with MRI is not helpful because it offers images that are difficult to differentiate from aggressive lesions. Hence, osteoid osteomas are identified mainly by images provided by plain radiographs or CT scans [9]. Holkar et al. compared the diagnostic accuracy of MRI compared to CT in a prospective study. They concluded that MRI was only 3% accurate, while CT imaging was 67% accurate [15].

The generic symptoms of osteoid osteomas can lead to a misdiagnosis. Foot osteoid osteomas may be mistaken for tibiotalar impingement syndrome. The latter is characterised by anterior ankle pain, joint oedema after activity, and occasionally reduced ankle dorsiflexion. The pain is caused by synovial tissue interference between the talus and the ankle mortise. In addition, the decreased tibiotalar arc of motion caused by spurs or osteophytes has also been implicated in this process. The diagnosis of anterior impingement is mostly based on clinical examination. Its most common symptom is local pain, exacerbated during palpation. Standard anteroposterior and lateral radiographs are required to detect the presence of osteophytes. However, the latter may not be identified radiographically due to the superposition of the more prominent anterolateral border of the distal tibia. The mainstay of treatment for tibiotalar impingement syndrome is activity modification, NSAIDs, and corticosteroid injections. Surgical management is indicated in patients with progressive symptoms who fail nonoperative management [16]. Similarly, the majority of osteoid osteomas are initially managed non-operatively with NSAIDs and regular follow-ups. The use of NSAIDs alleviates pain and assists in the improvement of symptoms. Hence, they are currently used as a stand-alone treatment for 50% of osteoid osteoma patients.

However, there are cases, such as osteoid osteomas of the distal phalanges, where the results of conservative treatment are not acceptable. In cases where conservative management fails, more invasive options are taken into consideration. These consist of open or arthroscopic surgical excision, as well as less invasive procedures like CT-guided percutaneous excision, radiofrequency ablation, laser photocoagulation, and MR-guided high-intensity focused ultrasound (MR-HIFU) [17]. During percutaneous radiofrequency ablation (RFA), a probe is driven under CT guidance to the selected lesion. It produces a thermal effect of 80-90 °C for six minutes, creating a necrotic zone of approximately 1 cm. The success rate of RFA after two sessions is 90%, while the recurrence rate is 10-15% [18]. The application of percutaneous radiofrequency ablation is relatively indicated for lesions that are adjacent to joints because the risk of cartilage damage is high. Furthermore, it is contraindicated for treating osteoid osteomas of the phalanges and those that develop proximal to nerve structures. Zouari et al. investigated the outcome of percutaneous CT-guided laser photocoagulation as an alternative treatment for patients with osteoid osteomas of the hands and feet [19]. They reported that most of their patients were pain-free within a week after their initial treatment and remained asymptomatic during follow-up. Moreover, no adverse events were documented after photocoagulation, leading the investigators to conclude that this treatment was effective and safe. Magnetic resonance-guided high-intensity focused ultrasound (MR-HIFU) is another alternative treatment for osteoid osteomas. It is a needle-free procedure of transcutaneous thermal ablation, causing periosteal neurolysis and coagulative necrosis of the nidus. It is less invasive than other treatment methods and offers good results regarding pain alleviation. Its application has been associated with a significant decrease in VAS score, Oswestry disability questionnaire score, and the brief pain inventory interference scale [20]. Surgical excision with curettage is reserved for lesions located close to the skin or near nerve tissue, as well as finger lesions and cases of painful scoliosis associated with osteoid osteoma. It is important to achieve a marginal resection of the nidus by either using a percutaneous, arthroscopic, or open approach. Regardless, the reported success rate spans up to 94%. However, when surgical resection is chosen, complications such as iatrogenic fractures, nerve or vessel injuries, and surgical site infections have been reported [14].

## Conclusions

Osteoid osteomas of the talus are rarely seen and therefore can be easily misdiagnosed. A high index of suspicion and an appropriate imaging examination are mandated for an early diagnosis. Computed tomography is the imaging modality of choice to investigate such entities. There are numerous treatment options, all of which offer good results when indicated. The surgical treatment of osteoid osteomas with curettage offers a success rate close to 97%.
